# Molecular Pathogenesis of Arrhythmogenic Cardiomyopathy: Mechanisms and Therapeutic Perspectives

**DOI:** 10.3390/biom15111512

**Published:** 2025-10-25

**Authors:** Eliza Popa, Sorin Hostiuc

**Affiliations:** 1 National Institute of Legal Medicine, 042122 Bucharest, Romania; eliza.popa@inml-mm.ro; 2 Department of Legal Medicine and Bioethics, Carol Davila University of Medicine and Pharmacy, 020021 Bucharest, Romania

**Keywords:** arrhythmogenic cardiomyopathy, molecular pathogenesis, gene therapy

## Abstract

Arrhythmogenic cardiomyopathy (ACM) is a genetic cardiac disease characterized by a progressive loss of cardiomyocytes associated with fibrofatty myocardial replacement, resulting in a heightened risk of ventricular arrhythmias and sudden cardiac death. ACM is a common cause of sudden death in young individuals, and exercise has been proven to be a factor in disease progression. Current therapeutic strategies, including lifestyle modification, antiarrhythmic pharmacological therapy, catheter ablation, and the placement of implantable cardioverter-defibrillators, remain primarily palliative options rather than addressing the underlying molecular substrate. The pathogenesis of ACM includes complex molecular and cellular mechanisms, linking genetic mutations to structural and electrical anomalies of the ventricle. The lack of targeted therapies contributes to a challenging approach to the disease. It highlights the need for a better understanding of the mechanisms that lead to myocardial remodeling and arrhythmic predisposition. With the help of animal models (especially murine) and induced pluripotent stem cells, there have been advances in understanding the molecular pathogenesis of ACM. In this review, we summarized some of the pathogenic molecular pathways involved in the development of ACM and emerging therapies targeted towards disease modification, not just prevention.

## 1. Introduction

Arrhythmogenic cardiomyopathy (ACM) is a hereditary disease characterized by a progressive loss of cardiomyocytes and their replacement with fibrous and fatty tissue [1]. The replacement of the healthy heart muscle with fibrofatty tissue creates a “myocardial scar,” leading to alteration of the typical architecture and electrophysiological properties of the heart, which results in regional or global ventricular dysfunction that predisposes patients to ventricular arrhythmias, irrespective of the systolic ventricular function [2].

ACM is a rare disease with a prevalence ranging from 1:2000 to 1:5000 individuals; however, it poses a significant risk factor for sudden cardiac death, especially among younger patients [3,4]. Even though it is considered a rare disease, the number of reported cases might be underestimated because the symptoms are often subtle or even concealed in their early phases [5]. The disease was initially described as arrhythmogenic right ventricular dysplasia (ARVD) before the involvement of the left ventricle was recognized, which led to the term ACM [6].

ACM is one of the main causes of sudden cardiac death (SCD) in young adults, especially in the under-35-years-old group. ACM holds significant importance in cases of exercise-related sudden deaths in athletes. SCD might be the first clinical presentation, suggesting the need for a better screening strategy oriented towards identifying high-risk groups [7,8]. James et al. demonstrated in their epidemiological study the link between exercise and the development of ACM. They suggested that intense physical activity and competitive sport participation can accelerate the progression of the disease and increase the risk of arrhythmia, while also increasing the likelihood of diagnosis [9]. The importance of physical activity as an environmental modifier for ACM has gained attention as it is one of the most common causes of sudden cardiac death in young individuals, especially in athletes [10]. There is no agreement regarding the effect of moderate physical activity or recreational sports, as some studies reported increased risk of arrhythmia, while others showed otherwise [11]. Studies on mice have shown that forced exercise protocols (running on a treadmill or swimming) can exacerbate ACM phenotypes, leading to increased fibrosis, reduced cardiac function, and an increased occurrence of arrhythmias or cardiomyocyte death [12,13]. At the same time, one study observed that, in some cases, low to moderate intensity voluntary exercise had a positive effect, suggesting the potential benefit of long-term light exercise [14]. Connexin-43 (Cx43) is a protein that is involved in cellular communication and cell-to-cell adhesion and is a component of gap junctions, allowing the passage between cells of ions and small molecules [15]. While Cx43 improves cardiac function, in ACM, there is a reduced expression and mislocalization of Cx43 at intercalated discs, contributing to impaired conduction and arrhythmogenesis [16]. Moderate exercise has been observed to increase Cx43 expression (in non-ACM models) [17,18], but high-intensity prolonged exercise tends to reduce Cx43 expression [19]. Taking this into account, the “Cx43 paradox” suggests that although enhancing Cx43 expression could be a therapeutic solution, excessive exercise can counteract the potential positive effects.

The clinical manifestations of ACM are most commonly caused by arrhythmias, with symptoms including palpitations, syncope, and cardiac arrest. Even though heart failure with its specific symptoms appears later in the course of the disease, for some patients, it may be the first sign [20]. The natural history for ACM usually consists of four phases: (i) the concealed phase in which there are no structural modifications, but the patients are still at risk, especially during strenous physical activity; (ii) the second phase is characterized by EKG abnormalities, like inverted T wave and arrhytmias with left bundle branch block; (iii) the third phase exhibiting the loss of healthy heart muscle that is being replaced by fibrous and fatty tissue that are usually detected by magnetic resonance; the end-phase is characterized by heart failure affecting both of the ventricles [21].

Conventional therapy strategies, such as implantable cardioverter-defibrillators (ICD), pharmacological options, or lifestyle changes, do not affect the molecular substrate that causes the disease to progress [22]. Recently, significant advances in molecular cardiology and genetics have provided insights into ACM pathogenesis. A consistent component of ACM pathogenesis involves genes that encode desmosomal proteins, resulting in the disruption of cell-to-cell adhesion and intracellular signaling [23]. By understanding the molecular mechanisms that define the disease, there is a better chance of an early diagnosis and an opportunity for novel therapeutic strategies.

This narrative review aims to integrate current knowledge of the molecular pathogenesis of ACM and explore new therapeutic perspectives that extend beyond traditional pharmacological and device-based treatments, moving towards approaches designed to alter the disease process itself.

## 2. Genetic Basis of ACM

The current classification of ACM consists of three subtypes, each defined by the preponderance of ventricular involvement [24]. The first and most common type is the right ventricle-dominant arrhythmogenic cardiomyopathy, formerly known as arrhythmogenic right ventricular dysplasia (ARVD) or arrhythmogenic right ventricular cardiomyopathy (ARVC) [25]. In this subtype, the right ventricle is dilated and exhibits focal wall motion anomalies. Commonly, the subtricuspid valve region and the right ventricular outflow tract are the most predisposed regions to remodeling, often leading to the formation of aneurysms [26]. The second type is the biventricular disease, with the involvement of both ventricles; this variant is associated with a greater risk of heart failure and earlier presentation of arrhythmia [27]. The third type refers to a more pronounced involvement of the left ventricle, known as arrhythmogenic left-ventricular cardiomyopathy (ALVC) or left-dominant arrhythmogenic cardiomyopathy, with no or minimal abnormalities of the right ventricle. Patients with ALVC have a higher incidence of ventricular arrhythmias, and episodes that can mimic myocarditis are common, with chest pain, troponin elevation, and ECG abnormalities [28]. The classification of ACM and the key clinical features are summarized in Table 1.

ACM is a familial disease in 30–50% of cases, usually with autosomal dominant transmission with variable penetrance [30]. It is now known to be a disease centered around the desmosome, with almost 90% of cases being linked to mutations in genes encoding desmosomal proteins [31]. Desmosomes are cell-to-cell junctions that anchor intermediate filaments to the plasma membrane [32]. Reports of familial clustering of ACM pointed to a genetic cause of the disease. By analyzing cases from the Naxos island in Greece, Protonotarios et al. observed that there were individuals who had ACM along with keratoderma and woolly hair [33]. Studies have pointed to two genetic causes for Naxos syndrome: mutations in genes that encode proteins involved in cell-to-cell adhesion—plakoglobin and desmoplakin [34]. Similarly to Naxos, Carvajal syndrome was reported in Ecuador, characterized by keratoderma, dry and blister-prone skin, woolly hair, and cardiomyopathy. Genetic studies also found a mutation in a gene that encodes desmoplakin [11]. While Naxos and Carvajal syndromes are autosomal recessive, most phenotypes of ACM have autosomal dominant transmission [21].

As stated previously, the known pathogenesis of ACM is usually centered around the desmosome. The most common desmosomal genes involved in ACM are PKP2 (encoding plakophilin-2), DSG2 (encoding desmoglein-2), DSC2 (encoding desmocollin-2), JUP (encoding plakoglobin), and DSP (encoding desmoplakin). Still, non-desmosomal protein involvement has also been reported, such as transmembrane protein 43 (TMEM43), desmin (DES), phospholamban (PLB), N-cadherin (CDH2), sodium voltage-gated channel alpha subunit 5 (SCN5A), titin (TTN), and transforming growth factor 3 beta (TGF3β) [21]. The classic form of ACM involving the right ventricle is usually caused by the mutation of the PKP gene [35]. The main genes involved in ACM are listed in Table 2.

## 3. Molecular and Cellular Pathogenesis

The pathogenesis of arrhythmogenic cardiomyopathy (ACM) consists of complex molecular and cellular mechanisms, linking genetic mutations to structural and electrical anomalies of the heart. These processes can be described as: desmosomal protein mutations with mechanical failure, dysregulation of signal transduction, programmed cell death, immune responses, and fibrofatty replacement of the cardiomyocytes. In this section, the processes that contribute to the pathogenic pathway leading to ACM are described.

### 3.1. Desmosomal Dysfunction

Desmosomes are specialized intercellular junctional structures, integrated into the intercalated discs of cardiomyocytes, with an essential role in maintaining the mechanical integrity of the cardiac muscle [52]. Three gene superfamilies contribute to the formation of desmosomes: cadherins, the armadillo family of nuclear and junctional proteins, and the plakins [23]. The cadherins are calcium-dependent adhesion molecules with multiple roles: embryogenesis, maintaining cell polarity, and signal transduction [53]. The desmosomal cadherins include two types of proteins encoded by separate genes: desmoglein (*DSG2)* and desmocollin (*DSC2*) [54]. The cardiac armadillo family consists of plakophilin-2 (*PKP2*) and junctional plakoglobin (*JUP*). They connect cadherins to desmoplakin and mediate the connection between desmoplakin and desmin, the intermediate filament [55]. The plakin family is represented by the desmoplakin (*DSP*), which connects the desmosome to the desmin [55].

Most genotype-positive cases have been linked to a genetic malfunction in at least one of the genes that encode the desmosomal proteins. Under mechanical strain, this compromised cell-to-cell adhesion causes cardiomyocytes to detach from one another, undergo apoptosis, and necrosis [56,57]. The repair mechanisms often trigger inflammatory pathways, fibrosis, and adipogenic pathways. In the end, these changes result in the hallmark of ACM: replacement of the ventricular myocardium with fibrous and fatty tissue [58,59].

### 3.2. Signal Transduction Pathways

#### 3.2.1. The Wnt/β-Catenin Signaling Pathway

**Figure 1:**
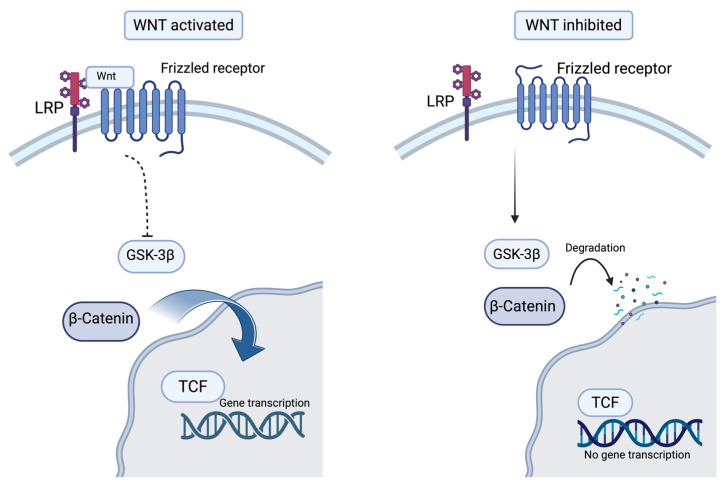
The Wnt/β-catenin pathway (Created in BioRender. Popa, E. (2025) https://BioRender.com/1vkur7t (accessed on 16 October 2025)).

The Wnt pathway is a signaling pathway involved in both embryo development and adult tissue homeostasis [60]. The canonical Wnt pathway, dependent on β-catenin, is activated when Wnt ligands attach to the Frizzled receptor and its co-receptor (lipoprotein receptor-related protein-LRP) [61,62]. The absence of Wnt stimulation leads to the phosphorylation of β-catenin, which is mediated by glycogen synthase kinase-3β (GSK-3β) and induces its degradation [62]. When Wnt ligands bind to the receptor complex, glycogen synthase kinase-3β (GSK-3β) activity is inhibited, resulting in the accumulation of β-catenin in the cytoplasm [62,63]. The β-catenin will translocate into the nucleus, where it interacts with TCF (T-cell factor) transcription factors, initiating signaling cascades [63]. In ACM, the inhibition of canonical Wnt/β-catenin has been associated with adipogenesis, fibrogenesis, and apoptosis [63,64,65]. As a structural relative of β-catenin, plakoglobin can interfere with β-catenin’s transcriptional activity by competing for binding, leading to degradation of β-catenin [11]. Desmosomal mutations increase the cytosolic plakoglobin (also known as γ-catenin), which can also be phosphorylated by GSK-3β and translocate into the nucleus, leading to the inhibition of β-catenin-mediated transcription and stimulating adipogenic signals [66]. Figure 1 illustrates a schematic representation of the Wnt/β-catenin pathway.

#### 3.2.2. Hippo Pathway Activation

**Figure 2:**
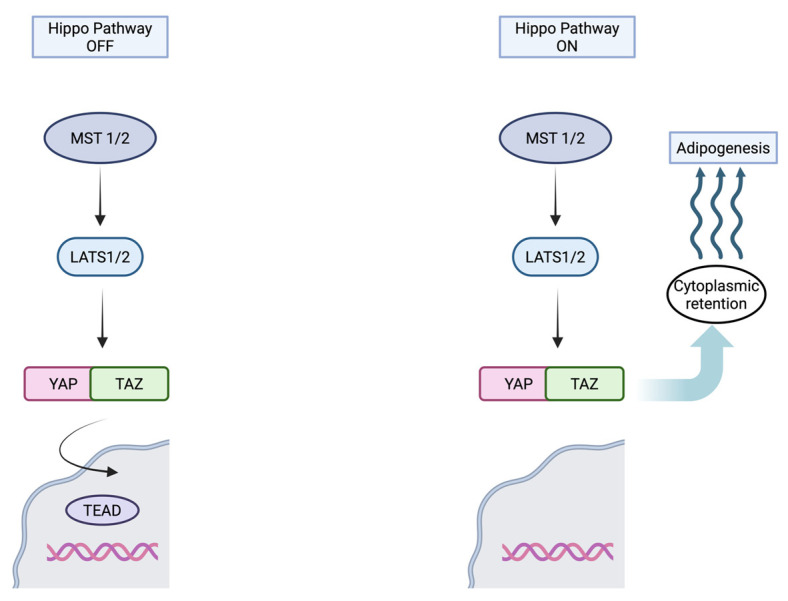
The Hippo Pathway (Created in BioRender. Popa, E. (2025) https://BioRender.com/ahfj5v4 (accessed on 16 October 2025)).

The Hippo pathway is a signaling pathway that controls organ size and tumor growth by regulating cell proliferation and apoptosis [67]. It is primarily composed of the kinases STE20-like protein kinase (MST), large tumor suppressor kinase (LATS1/2), and the transcriptional coactivators Yes-associated protein (YAP) and transcriptional coactivator with PDZ-binding motif (TAZ) [68]. YAP and TAZ are regulated negatively by the activation of the Hippo pathway [69]. When YAP and TAZ are dephosphorylated by inactive Hippo signaling, they translocate into the nucleus and interact with TEA domain (TEAD) transcription factor, leading to proliferation, differentiation, and apoptosis [63,70]. The remodeling process at the intercalated discs in ACM leads to abnormal activation of the Hippo pathway, resulting in increased cytoplasmic retention of phosphorylated YAP and TAZ levels, and changes in gene expression that promote adipogenesis and apoptosis [71]. The activation of the Hippo pathway also suppresses canonical Wnt signaling, further inhibiting YAP/TAZ activity, contributing to cardiomyocyte proliferation, differentiation, and apoptosis [71,72]. Figure 2 shows the components of the Hippo pathway.

#### 3.2.3. PPARγ Pathway

**Figure 3:**
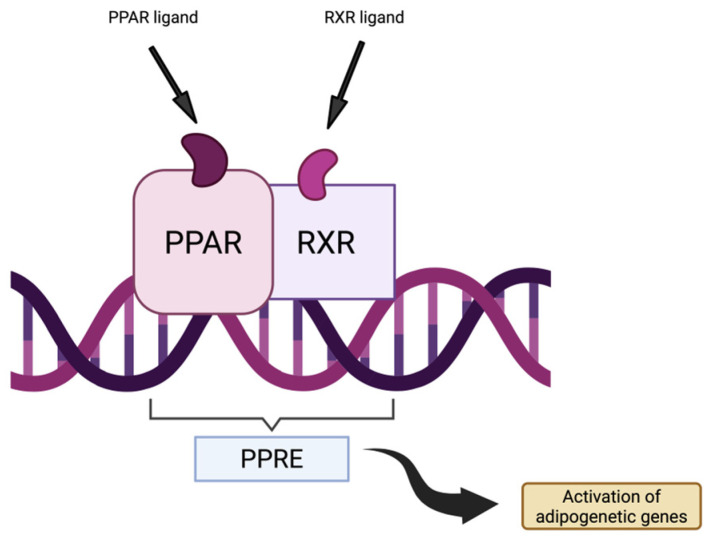
The PPARγ Pathway (Created in BioRender. Popa, E. (2025) https://BioRender.com/e3f7etr (accessed on 16 October 2025)).

Peroxisome proliferator-activated receptor gamma (PPAR*γ*) is a ligand-dependent transcription factor, part of the nuclear receptor family of peroxisome proliferator-activated receptors (PPARs) [73]. PPARs play an important role in adipogenesis, lipid metabolism, inflammation and overall metabolic homeostasis by forming a heterodimeric complex with retinoid X receptors (RXR), which can then bind to specific DNA sequences known as PPAR-responsive elements (PPREs) [74]. Overactivation of the PPARγ pathway has been involved as a downstream effector in ACM pathogenesis, as it contributes to the hallmark of fibro-fatty myocardial replacement [75,76]. Studies have observed in both human-induced pluripotent stem cell-derived cardiomyocytes and mouse models of ACM that an exaggerated activation of PPARγ leads to increased adipogenic differentiation and arrhythmogenic substrate formation [75,77]. Figure 3 illustrates the components mentioned above.

### 3.3. Cardiomyocyte Loss

While cardiomyocyte death plays a central role in the development of ACM, the precise mechanisms of cell death have not been fully identified [78]. Loss of cardiomyocytes can result from various causes, including apoptosis in programmed cell death pathways, necrosis, or cell injury, either from structural damage or inflammation-induced damage [11]. It has been indicated that programmed cell death is involved in cardiomyocyte loss in ACM [79]. In some studies, apoptosis had been associated with unusual transduction of mechanical signals [80,81,82]. In a survey conducted on neonatal rat ventricular myocytes by Hariharan et al., it was observed that, even though the overexpression of mutant plakoglobin did not affect cell adhesion, it promoted apoptosis through shear stress or cyclic stretch [80]. Another study conducted on human induced pluripotent stem cells with plakophilin-2 (*PKP2*) mutations, the authors showed evidence of increased apoptosis in cardiomyocytes through elevated rates of caspase-3 activation and mitochondrial cytochrome-c release. Also, treatment with apoptosis inhibitors, such as caspase blockers, minimized myocyte loss in cardiomyocyte cultures [81]. Moreover, desmoplakin suppression via small interfering RNA (siRNA) in cultured mouse atrial cardiomyocytes (HL-1 cardiomyocytes) and cardiac-specific desmoplakin (*DSP*) deficient mice led to upregulation of apoptotic markers and increased myocyte apoptosis [82]. Collectively, these findings suggest that apoptosis is a key pathogenic event in arrhythmogenic cardiomyopathy.

### 3.4. Inflammation and Immune Activation

Autopsy samples from patients with ARVC revealed that inflammatory infiltrates associated with severe structural changes are often present in the affected hearts [83,84]. Multiple studies have reported that inflammation is common in ACM hearts, especially in so-called “hot phases” that mimic myocarditis [85,86,87]. However, it is unclear whether inflammation is a primary event that leads to arrhythmia and damage to the myocardium or if it is a response caused by cardiomyocyte death [57]. A continuous activation of cardiac inflammatory pathways leads to fibrosis, resulting in myocardium remodeling [88]. Fibrosis creates a substrate for electrical and mechanical disturbances and reduces nutrient supply, resulting in a cycle of fibrosis, cardiomyocyte death, and inflammation [88,89]. A study by Lubos et al. used two murine models, one with mutant desmoglein 2 and one with absent desmoglein, to examine the inflammatory response during different phases of ARVC progression [90]. The results of this study suggested that inflammation in desmoglein-2 mutant mice is not only secondary to cardiomyocyte death [90]. In early phases, cardiomyocyte necrosis was observed with consequent neutrophil inflammatory response and upregulated chemokine pathways, showing that innate immune activation precedes significant structural remodeling [90]. Even in chronic phases, when fibrosis is already present, macrophages and T cells were still observed, suggesting that there is an inflammatory process that sustains disease progression [90].

A study by Asimaki et al. used neonatal rat ventricular myocytes to explore the link between inflammation and the desmosomal disruptions in ARVC [91]. They observed that even low concentrations of IL-17, TNF-α, and IL-6 led to the translocation of plakoglobin from intercellular junctions to intracellular sites, suggesting that inflammation is indeed involved in the pathogenesis of ARVC and can also drive the disease [91].

### 3.5. Adipogenesis

As noted, the replacement of healthy cardiomyocytes with fibrofatty tissue is the hallmark of ACM. The accumulation of adipose tissue occurs through actual adipogenic differentiation, resulting in the formation of mature adipocytes, as opposed to being a cellular accumulation of lipids [92]. However, even if adipogenesis is the central element of ACM, the exact origin and mechanisms remain unclear [93].

The main pathways that have been proposed to be involved in adipogenesis are Wnt, Hippo, and PPARγ [63,94]. Regarding the Wnt/β-catenin pathway, in a study by Garcia-Gras et al., using HL-1 cardiac myocytes with suppressed desmoplakin expression, they observed that, by destabilizing the desmosome, the plakoglobin was freed from the junctions and translocated into the nucleus, where it competes with β-catenin for binding to specific transcription factors, suppressing the canonical Wnt/β-catenin signaling [82]. Normally, Wnt activity inhibits adipogenic differentiation, so when it is suppressed, adipogenic genes are no longer inhibited [82]. The study also showed upregulation of PPARγ (peroxisome proliferator-activated receptor gamma), which is a known regulator of adipogenesis, and C/EBP-α (CCAAT/enhancer-binding protein-alpha), which cooperates with PPARγ in adipocyte differentiation [82,95,96]. To test the potential involvement of the Hippo pathway in adipogenesis, Chen et al. used samples from ACM patient hearts, mouse models (desmoplakin-deficient and plakoglobin-altered), and HL-1 cardiac myocytes with PKP2 knockdown [71]. Decreased PKC-α (protein kinase C alpha) activity with consequent activation of neurofibromin (an upstream molecule of the pathway), along with phosphorylation of downstream molecules such as MST1/2, LATS1/2, and YAP, inhibited Wnt/β-catenin, leading to initiation of adipogenic transcriptional program [71].

Initially, it was believed that the adipocytes derive from cardiomyocyte transdifferentiation. In a study by d’Amati et al., it was observed that transitional cells between cardiomyocytes and adipocytes expressed both vimentin, which is specific for muscular tissue, and desmin, which is specific for adipose tissue [97]. Another study that analyzed an ACM patient’s heart biopsy found an area in the ventricular myocardium, between a large amount of adipose tissue, where the cardiomyocytes exhibited abnormalities. They observed a change in the shape of the nucleus, perinuclear vacuolization, accumulation of granules resembling lipid droplets, and disruption of the plasma membrane, resulting in the discharge of intracellular contents into the interstitial space [98]. However, it is crucial to keep in mind that both of the studies were based on single cases. In an in vivo study, researchers found evidence that some adipocytes present in the hearts of desmoplakin knockout mouse models originate directly from cardiomyocytes. These adipocytes were located in the sub-epicardial region, except the midwall, and this conclusion was drawn by using the ventricular isoform of myosin light chain 2 (MLC2v) labeling on the cardiomyocytes [99]. Dorn et al. proposed the presence of shared progenitor cells in the normal heart that have the potential to differentiate into either cardiomyocytes or adipocytes. These cells were identified by the expression of Isl1 (ISL LIM Homeobox 1) and Wt1 (Wilms tumor 1). Depending on the stimuli, they could be pushed or biased towards an adipogenic fate, leading to the fatty replacement that characterizes ACM [100]. The epicardium, the outermost layer of the heart, is composed of mesothelial cells that are usually inactive in the adult heart, unless there is a response to a disease. In the context of development or disease, epicardial cells can transition to mesenchymal cells, allowing them to differentiate into various cardiac cell types [101]. Given this and the fact that fibro-fatty tissue is predominant in the sub-epicardial area in ACM hearts, the epicardial cells have been proposed as a potential source for the fibro-fatty tissue [94].

### 3.6. Arrhythmogenesis

As noted, the fibrofatty replacement of the normal ventricular tissue is the main characteristic of ACM, creating a substrate that is highly prone to life-threatening arrhythmias. Notably, even in earlier phases, when the ventricle appears to be structurally normal, the risk of arrhythmias is still present [102].

Along with desmosomes, other adhesion junctions, gap junctions, and sodium channels reside at the intercalated disk [103]. While desmosomes provide mechanical support for the intercalated disks, they also maintain the proper function of ion channels located in cardiac intercalated discs [104]. The cardiac sodium channel Na(v)1.5 is an essential component for the initiation and conduction of action potentials, therefore responsible for the regular rhythm in the heart [105]. It has been observed that there is a tight connection between the desmosomal protein complex and the cardiac sodium channel, which explains the early arrhythmias observed in patients with no or limited altered structure at a macroscopic level [102]. In their study, Rizzo et al. noted that this happens because of a reduction in action potential upstroke velocity consequent to a decrease in cardiac sodium current density [102]. The cardiac sodium channel had been previously associated with components of the desmosome, specifically plakophilin-2 [106]. A study has noted that loss of PKP2 expression was associated with reduced peak current density, negatively shifted voltage-dependence of inactivation, and prolonged recovery from inactivation of sodium currents [106]. A study also investigated the prevalence and functional implications of the sodium channel mutations in patients without known desmosomal mutations [48]. The authors performed whole-exome sequencing on six patients and found a rare missense variant (R1898H) [48]. The sodium channels and currents were studied in R1898H human induced pluripotent stem cell-derived cardiomyocytes (R1898H hiPSC-CM). Patch-clamping revealed a 36% reduction in peak sodium current compared to corrected controls, and fluorescence microscopy demonstrated decreased sodium channels and N-cadherin clusters at the intercalated disc [48]. This study also suggests that the sodium channel is part of a complex with adhesion molecules, like N-cadherin. Disrupting this complex may impair sodium current and intercalated disk integrity.

Connexin 43 (Cx43) is the predominant gap junction protein in ventricular myocardium, forming low-resistance electrical channels that allow rapid propagation of action potentials between cardiomyocytes [107]. Cx43 is localized at intercalated discs, where it forms hexameric connexons that attach to neighboring cells to form gap junctions [108]. The phosphorylation state and precise organization of Cx43 are essential for maintaining normal cardiac rhythm [109]. In ACM, the mutations in genes encoding desmosomal proteins cause secondary remodeling of gap junctions and reduced expression of Cx43, even in histologically unaffected regions of the miocardium [23]. Reduced expression and altered localization of Cx43 have been demonstrated both in human samples and experimental models. Oxford et al. reported a decreased expression and a redistribution of Cx43 in PKP2 knockdown rat cells, with Cx3 shifting from the plasma membrane contact zones to a more intracellular localization [110]. Asimaki et al. performed immunohistochemical analysis of human myocardial samples from patients with ACM. They observed that the biopsy samples from all subjects had a reduction in the amount of junctional signal for connexin 43 [111].

## 4. Current Clinical Management

ACM should be suspected particularly in younger individuals who present symptoms such as palpitations, syncope, or aborted sudden death [112]. The main goals of clinical management for patients with ACM are to improve symptoms by slowing the progression of structural remodeling and preventing complications such as ventricular tachycardia, development of heart failure, or sudden cardiac death [113].

Lifestyle and activity modifications are essential; it is strongly recommended that patients with ACM avoid intense and competitive sports, as prolonged and strenuous physical activity has been shown to increase the risk of ventricular arrhythmias and sudden cardiac death [114].

Pharmacological therapy is currently the cornerstone of disease management, with beta-blockers, amiodarone, sotalol, and flecainide being the preferred treatments [60]. Among these, beta-blockers are the first choice because they reduce adrenergic stimulation. This approach is especially beneficial during exercise. Titrating to the maximum tolerated dose of beta-blockers has been associated with increased survival from ventricular arrhythmias [115]. If beta-blockers are ineffective to control ventricular extrasystoles or tachycardia, additional antiarrhythmic agents such as amiodarone or flecainide can be added [116].

In ACM, the fibrofatty tissue replacing healthy myocardium creates a highly arrhythmogenic substrate, predisposing patients to sustained ventricular tachycardia and ventricular fibrillation. When pharmacological therapy fails to control recurrent arrhythmias adequately, catheter ablation can be an option [113]. Modern ablation strategies often use three-dimensional electroanatomical mapping to identify scar regions and abnormal conduction pathways, enabling a more complex endocardial and epicardial approach. Still, this method is associated with a high recurrence rate [113].

Another option that can be used if pharmacological therapy cannot control the ACM symptoms is the implantation of a cardioverter defibrillator. The implantable cardioverter defibrillator is usually recommended for secondary prevention of sudden cardiac death [60]. The indication for primary prophylactic implantation in patients with ACM remains debatable due to the lack of large randomized clinical trials that provide clear evidence of benefit [116]. Unlike secondary prevention, where a life-threatening arrhythmia has been established and then the placement of an ICD has been decided, the criteria for primary prevention rely on individual risk assessment models. The ARVC risk calculator has become a standard for predicting the 5-year risk of life-threatening arrhythmias and guiding the ICD placement decision [116,117]. According to the European Society of Cardiology (ESC) 2023 guidelines [60], ICD implantation is strongly recommended (Class I) for patients with ACM who have survived cardiac arrest or experienced sustained ventricular tachycardia associated with syncope, as these are clear markers of high risk for sudden death. Class IIa recommendation suggests that ICD should be implanted in patients who have sustained ventricular tachycardia without syncope but have other high-risk clinical features such as arrhythmic syncope, non-sustained ventricular tachycardia (NSVT), right ventricular ejection fraction (RVEF) < 40%, left ventricular ejection fraction (LVEF) < 45%, or sustained monomorphic ventricular tachycardia (SMVT) during programmed electrical stimulation (PES) [60].

## 5. Emerging Therapeutic Approaches

Actual treatment for ACM focuses on symptom control, arrhythmic risk reduction, and prevention of sudden cardiac death [60]. Unfortunately, current therapeutic options for ACM have seen little evolution and often have limited success. While lifestyle changes, along with the pharmacological and invasive approaches mentioned above, can improve patient outcomes, they remain largely palliative and frequently insufficient, especially in severe forms of the disease [118]. Recent studies have provided a somewhat enhanced understanding of the molecular pathogenesis of ACM, with opportunities for more targeted therapies. However, these emerging therapeutic options are still in early stages and lack evidence of their long-term safety.

### 5.1. Gene Therapy

Gene replacement therapy consists of the delivery of a healthy transgene to the target organ, usually using a viral vector [118]. Currently, the most commonly used and efficient viral vectors are derived from retroviruses, lentiviruses, adenoviruses, and adeno-associated viruses (AAV) [119]. The adeno-associated virus (AAV) is a small, non-pathogenic parvovirus with a non-enveloped protein capsid that carries single-stranded DNA [120]. Among these viral vectors, recombinant adeno-associated viruses (rAAV) are the leading option for delivering genes to the heart due to their high efficiency in gene transfer, low immune response, and unique capsid-dependent tissue tropism [121]. The capsid of the AAV has a vital role in determining vector tropism and influences both the efficiency of receptor-mediated entry and the level of transgene expression [120].

The *PKP2* gene has been the focus of most preclinical studies, mainly based on its high prevalence in clinical cohorts [121]. Specifically, a deficit of the *PKP2* gene leads to reduced contractility, disruption of the intercalated disc structure, and damaged desmosomal assembly [122].

Bradford et al. utilized the recombinant adeno-associated (rAAV) vector to deliver a functional copy of the plakophilin-2 (*PKP2*) gene to the ventricular cardiac tissue of a mouse model with ARVC [123]. They administered AAV9-PKP2 (serotype 9 of adeno-associated virus that carries a copy of the plakophilin-2 gene) to mice with established ACM disease and observed a notable decrease in arrhythmic events, improved cardiac function, and even partial reversal of fibrofatty infiltration. The authors used a knock-in mouse model that carries a *PKP2* splice-site mutation. Homozygous mutants exhibited sudden death starting at approximately 4 weeks, with a median survival time of 11 weeks, and no mice survived beyond 26 weeks. The homozygous mice displayed early onset of arrhythmias, a prolonged QRS complex, ventricular dysfunction, fibrofatty myocardial replacement, desmosomal protein loss, and inflammation. The administration of AAV9-PKP2 in young mice, before the onset of disease symptoms, led to a reduction in arrhythmic events, improved cardiac function, and preserved myocardial structure. In early therapeutic intervention, treated *PKP2* homozygous mice had 100% survival up to 6 months. By administering AAV-PKP2 when the disease is already established, there were still some improvements, such as the restoration of desmosomal proteins, reduced arrhythmias, and decreased fibrosis, fatty replacement, and inflammation. 100% survival was observed at 20 weeks in this case. Regarding the safety of AAV-PKP2 delivery, the mice showed no liver toxicity after long-term use, with normal liver enzyme levels [123].

Another study, by Kyriakopoulou et al., also used an AAV-mediated delivery of a functional *PKP2* gene [124]. They used induced pluripotent stem cell (iPSC)-derived cardiomyocytes with *PKP2* c.2013delC/WT haploinsufficiency to evaluate efficacy and corresponding in vivo mouse models. AAV6 vectors expressing wild-type human *PKP2* (AAV6-PKP2) managed to restore *PKP2* protein levels and normalize related junctional proteins like *JUP* and *DSP*, while *DSC2* and *DSG2* remained unchanged. Whole-cell patch-clamp recordings measured the sodium conduction to evaluate the peak transient sodium current density. These authors also reported an abnormal sodium conduction that, with treatment, was normalized at a level comparable with healthy controls. Since *PKP2* plays an essential role in myocyte adhesion and interaction, the authors created a three-dimensional engineered human myocardium containing 70% iPS-cell-derived cardiomyocytes and 30% human foreskin fibroblasts in a hydrogel made of type I collagen. The engineered human myocardium had reduced contractile force and kinetic anomalies (regarding beating frequency, contraction velocity, and relaxation velocity). With AAV6-PKP2 treatment, contraction amplitude was improved and kinetics were normalized (elongation of contraction and relaxation), showing excellent results by day 42 of treatment. Mice that carry the murine equivalent of the human *PKP2* mutation received the treatment intraperitoneally in 5-day-old pups and intravenously in adults. Improved desmosomal protein levels were observed, particularly in *JUP*, *DSP*, and *DSG2*, while *DSC2* remained unchanged, similar to the in vitro findings. Mice treated at 2 months with a single dose were followed up to 12 months, showing restored desmosomal integrity and prevention of diastolic dysfunction. *PKP2* gene replacement showed great promise, as it not only restored the primary protein but also other junctional proteins, producing long-lasting results without side effects in healthy models [124].

Wu et al. also determined the efficacy of adeno-associated virus 9 (AAV9) vector in restoring *PKP2* expression in mouse models [125]. The authors engineered a tamoxifen-inducible, cardiac-specific *PKP2* knockout mouse to induce ARVC. They also tested in vitro human induced pluripotent stem cell-derived cardiomyocytes (iPSC-CMs) with *PKP2* silencing. One dose was administered intravenously to the mice, either before disease onset or after the disease was already established. In vitro, *PKP2* silencing resulted in down-regulation of desmosome, sarcomere, and ion-channel gene expression with consequent impaired contraction and electrical signaling. In vivo, those mice developed arrhythmias, dilation of the right ventricle, reduced ejection fraction, remodeling, and rapid mortality (weeks). The results indicate that a single dose can prevent the development of cardiomyopathy before the onset of the disease and prolongs median survival by up to 58 weeks compared to untreated mice, where the median survival was approximately 4.7 weeks. When the cardiomyopathy has developed, it can slow its progression. There was also an extended median survival to >50 weeks. By delivering a functional *PKP2* gene, the reduction in the left ventricular ejection fraction was prevented, the frequency of ventricular arrhythmias was lower, and the dilation of the right ventricle was either prevented or even reversed. RNA sequencing showed a mass-normalization of gene expression across desmosome, sarcomere, ion channel, inflammatory, apoptotic, and fibrosis pathways. The U.S. Food and Drug Administration has approved the Investigational New Drug (IND) application, allowing the beginning of clinical trials for this new therapy [125].

Another study by Opbergen et al. investigated the effects of a gene therapy using an AAVrh.74 vector (AAVrh.74-PKP2a, also designated RP-A60) that delivers a functional human PKP2 transcript to mice with tamoxifen-induced, cardiomyocyte-specific *PKP2* knockout [126]. A single dose was administered via tail-vein injection, and after 28 days, tamoxifen was given to induce *PKP2* knockout. Some of the mice received the treatment dose after disease onset, at 7 or 14 days after the administration of tamoxifen. *PKP2* levels were increased after treatment, and immunofluorescence studies showed *PKP2* localization at the intercalated discs. Treated mice also showed a decrease in myocardial fibrosis, as well as smaller collagen deposits in the ventricles. While untreated mice had 100% mortality at about 50 days after tamoxifen induction, the ones that received the gene therapy survived beyond 5 months. This relatively late administration (at 7 and 14 days after receiving tamoxifen) in the setting of established disease, fielded promising results with increased survival and preserved cardiac function [126].

The results of these studies suggest that using a viral vector to deliver the *PKP2* gene holds great promise for the future treatment of patients with ACM, especially those with *PKP2* mutations.

### 5.2. Current Clinical Trials

At present, three human clinical trials are recruiting to evaluate this new possible therapy: HEROIC-PKP2 (identifier: NCT06109181), RIDGE-1 (identifier NCT06228924), and RP-A601-0323 (identifier NCT05885412) [118]. The participants will receive a single dose administered intravenously and will be monitored for the next five years. All three studies will evaluate modifications in *PKP2* expression through cardiac biopsy. Results from these trials could determine if gene therapy can become a real disease-modifying treatment option, potentially changing the course of a condition that currently benefits only from palliative approaches.

The clinical trial for HEROIC-PKP2 (identifier: NCT06109181) by Lexeo Therapeutics is still ongoing, but their most recent year-end 2024 report has already highlighted some promising results. Six participants enrolled in the trial, divided into two cohorts. Post-treatment cardiac biopsies were obtained from two of the participants in cohort 1, while the third participant decided not to undergo the biopsy procedure. The biopsies from the first two participants showed increased *PKP-2* protein expression, measured using Western blot assay. Increases of 71% and 115% were observed in *PKP-2* protein expression compared to the pre-treatment baseline. The first participant who reached six months post-treatment showed a 67% reduction in premature ventricular contractions from baseline and normalized QRS duration. The report mentions that the treatment has been well-tolerated with no reports of serious adverse events [127].

Thenaya Therapeutics has already enrolled the first three participants at a dose of 3 × 10^13^ vg/kg in the RIDGE-1 Phase 1b trial of TN-401 (identifier NCT06228924). In July 2025, the trial’s Data Safety Monitoring Board (DSMB) reviewed the available data and approved dose escalation to 6 × 10^13^ vg/kg (Cohort2), along with the expansion of Cohort1 enrollment. At the moment, the first of three participants in the higher dose cohort has received the gene therapy. Currently, additional data and outcomes from the trial have not yet been reported [128].

Rocket Pharmaceuticals presented their preliminary data from Phase 1 of the clinical trial of RP-A601-0323 (identifier NCT05885412) at the 28th Annual Meeting of the American Society of Gene and Cell Therapy. Preliminary data show that in cardiac biopsies of three patients with ACM, there was increased PKP2 protein expression. Two of the participants with a low baseline level of PKP2 have an increase in the protein by 110% and 398% at the six-month follow-up. RP-A601 also increased protein expression and promoted desmosomal localization of Desmocollin-2 and Cadherin-2 in all three patients. There are also some notes on stabilization or improvement in cardiac function such as: maintained normal proper ventricular function in all patients, improved KCCQ-12 scores by 34–41 points and improved NYHA class from II to I in patients followed beyond 6 months, reduction in premature ventricular contractions by 9–63%, complete resolution of non-sustained ventricular tachycardias at 6 months, decrease from six to two leads of T-wave inversions in one patient at 6 months. At the moment, the tested therapy is well tolerated, with no dose-limiting toxicities observed in patients followed up to 12 months, while most adverse events were mild/moderate, with only one patient experiencing a more severe adverse reaction that was solved within two months, likely resulting from the immunomodulation regimen [129].

### 5.3. Targeted Molecular Therapies

As we mentioned above, the canonical Wnt/β-catenin pathway is crucial for myocyte differentiation and tissue integrity and is involved in ACM pathogenesis. Glycogen synthase kinase 3 beta (GSK-3β) is a serine/threonine kinase that is engaged in the regulation of the canonical Wnt signaling pathway [130]. Given GSK-3β’s central role in the downregulation of Wnt signaling, the inhibition of the kinase has been proposed as a possible therapeutic option in ACM [31]. By investigating a GSK-3β inhibitor, SB2, researchers reported the involvement of dysregulated GSK-3β signaling. The SB2 occupies the ATP-binding site of the kinase, blocking it from phosphorylating downstream substrates [131,132]. Lithium is known for its inhibiting properties on the GSK-3β [133]. Accordingly, in a study by Hamstra et al., low doses of lithium were given to wild mice for 6 weeks to inhibit the kinase. The effects on the cardiac SERCA (sarco/endoplasmic reticulum calcium ATPase) function compared with control-fed mice [134]. SERCA uses the energy from ATP hydrolysis to move cytosolic calcium against its concentration gradient into the sarcoplasmic reticulum, resulting in muscle relaxation [135]. Lithium-fed mice showed reduced GSK3β activity compared to control-fed mice and an increased calcium affinity of SERCA. While therapeutic doses of lithium that are commonly used in psychiatry have significant toxicity, this study indicated that a low-dose lithium, which inhibits GSK3β, could improve SERCA pump activity. It thus may be a potential therapy option to improve heart failure and cardiomyopathy [134]. The work of Chelko et al. demonstrated that SB2 can protect against myocyte damage and cardiac dysfunction in vivo in two murine models of ACM [105]. In disease-free hearts, GSK3β is located diffusely in the cytoplasm, but the authors of the study observed that in the ACM mice, the kinase accumulates intracellularly near intercalated discs. In mice with desmosomal mutations, the kinase inhibitor reduced ventricular ectopy, fibrosis and inflammation compared with vehicle-treated controls [136]. SB2 improved left ventricular function and survival rate in mice with a homozygous mutation of desmoglein-2 (*DSG2*), both in sedentary conditions and in exercise. This kinase inhibitor also restored the normal localization of intercalated disc proteins, such as plakoglobin, connexin43 and SAP97 [136]. While these findings underline the central role of GSK3β signaling, they also reveal a promising therapeutic option with the use of SB2.

## 6. Future Directions

Current clinical management of arrhythmogenic cardiomyopathy remains palliative mainly, focusing on symptom control and prevention of life-threatening arrhythmia through lifestyle changes, anti-arrhythmics and implantable devices.

The future of ACM therapy lies in strategies capable of disease modification by directly addressing the underlying molecular substrate. While gene therapy targets the primary genetic defect, the dysregulated signaling pathways, specifically Wnt/β-catenin/GSK-3β, present potential targets for future treatment to stop the progression of myocardial damage.

The most promising avenue for a curative approach seems to be gene therapy, particularly for the most common genotype associated with PKP2 mutations. Preclinical studies utilizing recombinant adeno-associated viruses to deliver a functional copy of the PKP2 gene have shown remarkable results. They demonstrate the capacity to prevent disease onset when administered early or even partially reverse established disease by restoring desmosomal integrity [123,124,125,126]. These encouraging preclinical findings provided the rationale for advancing PKP2 gene therapy into clinical trials. As of late 2024/early 2025, gene therapy candidates are being evaluated in human clinical trials and overall, early-phase clinical trials of gene therapies targeting PKP-2-related arrhythmogenic cardiomyopathy show promising results [127,128,129]. From the information currently available regarding the ongoing studies, therapies have consistently increased PKP-2 protein expression in cardiac tissue, resulting in reduced arrhythmias and improved ventricular function. So far, the gene therapy has generally been well-tolerated with few adverse events and no dose-limiting toxicities observed. However, these clinical studies are in the early phases, and further follow-up and additional data are needed to confirm long-term safety and true efficacy, as well as to determine the optimal dosage.

Although gene therapies present a potential cure and significant progress has been made in their development, current trials are largely limited to the PKP-2 genotype. In contrast, other common ones (DSP, DSG2) remain unaddressed. Furthermore, strategies to treat advanced disease, where fibrofatty replacement is already established, remain unexplored. Given the efficacy of gene therapy in disease prevention, identifying at-risk individuals in the concealed phase is crucial. Future research should therefore be focused on improving screening methods to enable earlier detection and intervention.

## 7. Conclusions

ACM is a multifaceted cardiac disease linked to genetic and molecular disturbances that end in structural and electrophysiological abnormalities. While conventional treatment focuses on prevention, it does not stop disease progression. Advances in molecular cardiology have discovered plausible new therapeutic targets, paving the way for a disease-modifying approach. Gene therapy using viral vectors to deliver the normal *PKP2* gene and GSK-3β inhibitors has shown excellent results in preclinical studies on animals, with preservation of myocardial structure and function. Current clinical trials are still ongoing with promising but minimal data at the moment. A more proper understanding of ACM’s molecular mechanisms could provide therapies that not only prevent the disease but also modify its trajectory.

## Figures and Tables

**Table 1:** Classification of ACM and key clinical features—based on [25,27,29].

ACM Variant	Predominant Ventricular Involvement	Key Clinical Features
ARVC	Right ventricle	RV fibro-fatty replacement, ventricular arrhythmias, typical onset in adolescence or early adulthood.
BiV-ACM	Both ventricles	Early LV involvement alongside RV, higher risk of heart failure.
ALVC	Left ventricle	LV arrhythmias, subepicardial fibrosis, and misdiagnosis as DCM are common.

ARVC = Arrhythmogenic Right Ventricular Cardiomyopathy; BiV-ACM = Biventricular Arrhythmogenic Cardiomyopathy; ALVC = Arrhythmogenic Left Ventricular Cardiomyopathy; RV = Right Ventricle; LV = Left Ventricle; DCM = Dilated Cardiomyopathy.

**Table 2:** Genes involved in ACM.

Genes	Protein Encoded	Evidence Level
PKP2	Plakophilin-2	Strong [36,37,38,39]
DSG2	Desmoglein-2	Strong [38,40]
DSC2	Desmocollin-2	Strong [38,40,41]
JUP	Plakoglobin	Strong [38,42]
DSP	Desmoplakin	Strong [38,43]
TMEM43	Transmembrane protein 43	Strong [38,44]
DES	Desmin	Moderate [38,45]
PLB(PLN)	Phospholamban	Moderate [38,46]
CDH2	N-cadherin	Limited [38,47]
SCN5A	Sodium voltage-gated channel alpha subunit 5	Limited [38,48]
TGF3β	Transforming growth factor 3 beta	Limited [38,49]
LMNA	Lamin A/C	Limited [38,50]
FLNC	Filamin C	Not defined yet [38,51]

## Data Availability

Not applicable.

## Abbreviations

The following abbreviations are used in this manuscript:

AAV	Adeno-Associated Virus
AAV6	Adeno-Associated Virus, Serotype 6
AAV9	Adeno-Associated Virus, Serotype 9
ACM	Arrhythmogenic Cardiomyopathy
ALVC	Arrythmogenic Left Ventricular Cardiomyopathy
ARVC	Arrhythmogenic Right Ventricular Cardiomyopathy
ARVD	Arrhythmogenic Right Ventricular Dysplasia
β-catenin	Beta-Catenin (Transcription Factor)
C/EBP-α	CCAAT/Enhancer-Binding Protein Alpha
Cx43	Connexin-43
CDH2	N-Cadherin
DCM	Dilated Cardiomyopathy
DES	Desmin
DSC2	Desmocollin-2
DSG2	Desmoglein-2
DSP	Desmoplakin
EKG	Electrocardiogram
ESC	European Society Of Cardiology
FDA	Food and Drug Administration
GSK-3β	Glycogen Synthase Kinase 3 Beta
ICD	Implantable Cardioverter-Defibrillator
IND	Investigational New Drug
IL-1β	Interleukin 1 Beta
IL-6	Interleukin 6
iPSC	Induced Pluripotent Stem Cells
iPSC-CM	Induced Pluripotent Stem Cell-Derived Cardiomyocyte
ISL1	ISL LIM Homeobox 1
JUP	Junctional plakoglobin
LATS1/2	Large Tumor Suppressor Kinase 1/2
LVEF	Left Ventricular Ejection Fraction
LV	Left Ventricle
MLC2v	Myosin Light Chain 2 (ventricular isoform)
MST1/2	Mammalian STE20-like Protein Kinase
NSVT	Non-Sustained Ventricular Tachycardia
PES	Programmed Electrical Stimulation
PKC-α	Protein Kinase C Alpha
PKP2	Plakophilin-2
PLB	Phospholamban
PPARγ	Peroxisome Proliferator-Activated Receptor Gamma
rAAV	Recombinant Adeno-Associated Virus
RVEF	Right Ventricular Ejection Fraction
RV	Right Ventricle
SAP97	Synapse-Associated Protein 97
SCN5A	Sodium-Voltage-Gated Channel Alpha Subunit 5
SB2	Selective GSK-3β Inhibitor
SCD	Sudden Cardiac Death
SERCA	Sarco/Endoplasmic Reticulum Calcium ATPase
siRNA	Small Interfering RNA
SMVT	Sustained Monomorphic Ventricular Tachycardia
TAZ	Transcriptional Coactivator with PDZ-binding Motif
TCF	T-cell Factor
TEAD	TEA Domain Family Member
TGF-β	Transforming Growth Factor Beta
TGF3β	Transforming Growth Factor 3 Beta
TMEM43	Transmembrane Protein 43
TNF-α	Tumor Necrosis Factor Alpha
TTN	Titin
WT1	Wilms Tumor 1
Wnt	Wingless-related integration site (signaling pathway)
YAP	Yess-associated Protein
